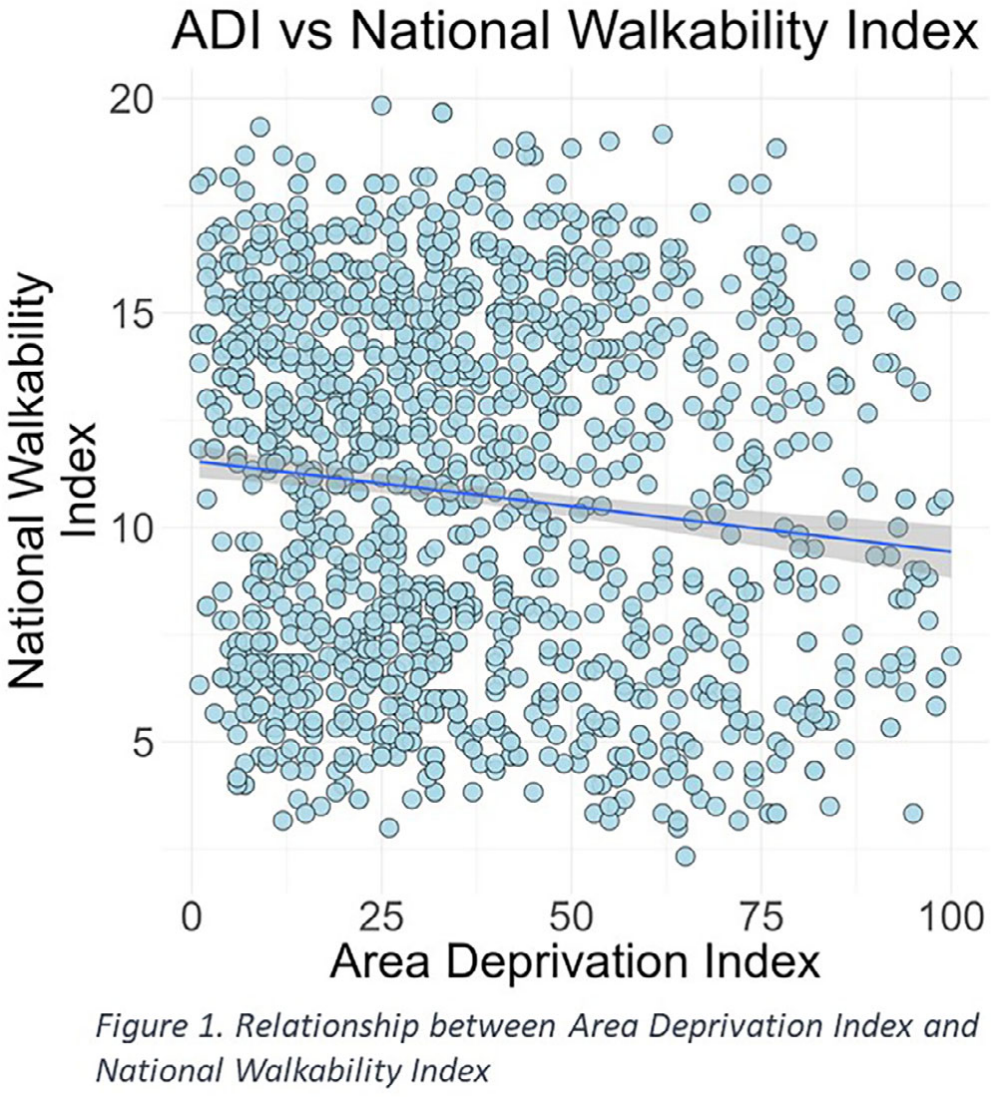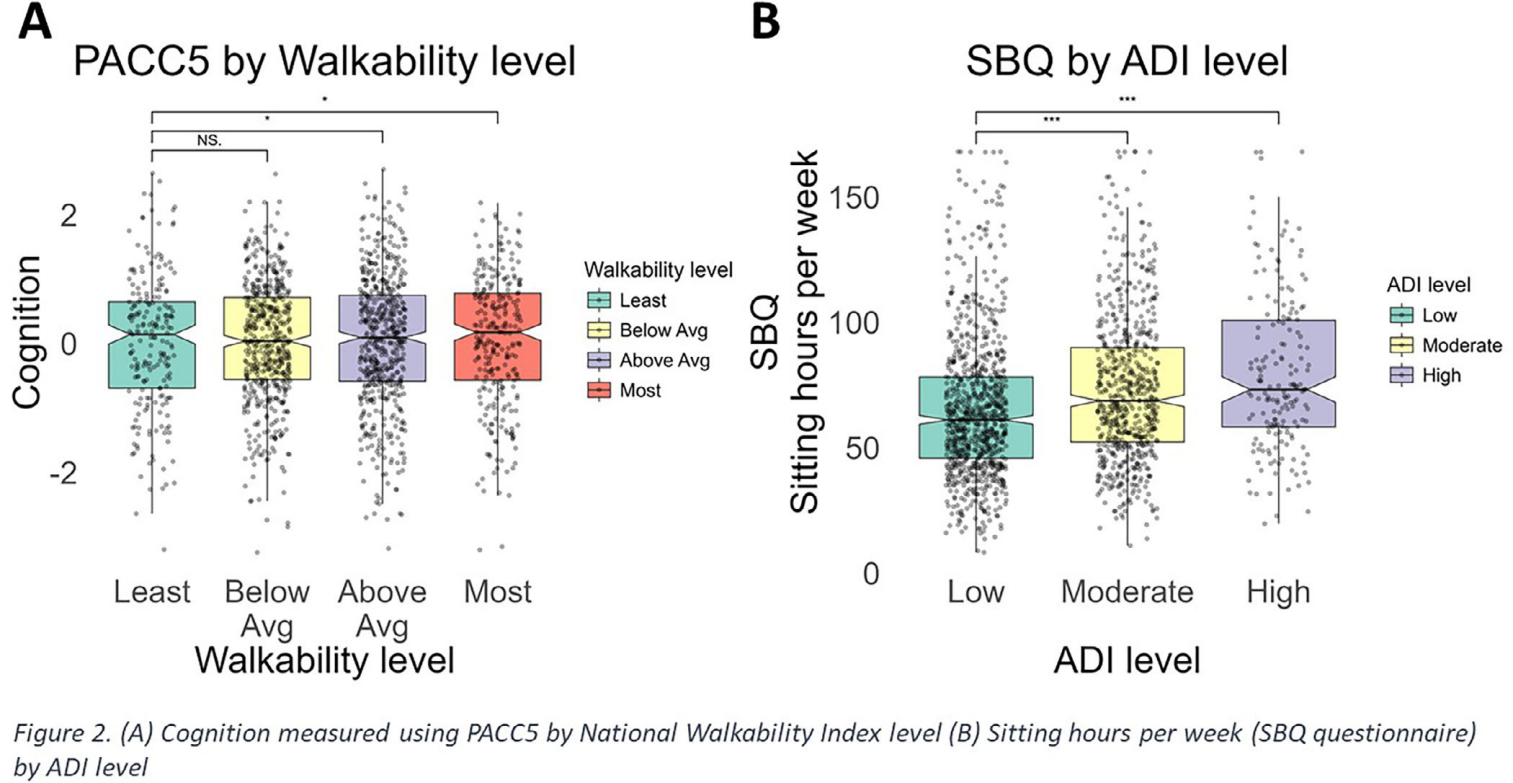# Association of neighborhood social and built environment with physical activity in U.S. POINTER

**DOI:** 10.1002/alz70860_105496

**Published:** 2025-12-23

**Authors:** Pablo Aguilar, Thomas Monroe Holland, Sam N. Lockhart, Joseph C. Masdeu, Lycia Tramujas Vasconcellos Neumann, Antonia Valentín, Sami Petricola, Judith Garcia‐Aymerich, Mark Nieuwenhuijsen, Heather M Snyder, Laura D Baker, Susan M. Landau, Eider M Arenaza‐Urquijo

**Affiliations:** ^1^ Global Health Institute Barcelona (ISGlobal), Barcelona, Spain; ^2^ University of Pompeu Fabra (UPF), Barcelona, Spain; ^3^ Sant Pau Memory Unit, Department of Neurology, Hospital de la Santa Creu i Sant Pau, Institut d'Investigació Biomèdica Sant Pau (IIB SANT PAU), Facultad de Medicina ‐ Universitat Autònoma de Barcelona, Barcelona, Spain; ^4^ Rush Institute for Healthy Aging, Chicago, IL, USA; ^5^ Rush University Medical Center, Chicago, IL, USA; ^6^ Wake Forest University School of Medicine, Winston‐Salem, NC, USA; ^7^ Houston Methodist Research Institute, Houston, TX, USA; ^8^ Alzheimer's Association, Chicago, IL, USA; ^9^ Department of Experimental and Health Sciences, Universitat Pompeu Fabra (UPF), Barcelona, Spain; ^10^ Centro de Investigación Biomédica en Red de Epidemiología y Salud Pública (CIBERESP), Madrid, Spain; ^11^ ISGlobal, Barcelona Institute for Global Health ‐ Campus MAR, Barcelona Biomedical Research Park, Barcelona, Spain; ^12^ Neuroscience Department, University of California, Berkeley, Berkeley, CA, USA; ^13^ Mayo Clinic, Rochester, MN, USA; ^14^ ISGlobal ‐ Barcelona Institute for Global Health, Barcelona, Catalunya/Barcelona, Spain; ^15^ Centro de Investigación Biomédica en Red de Fragilidad y Envejecimiento Saludable (CIBERFES), Instituto de Salud Carlos III, Madrid, Spain

## Abstract

**Background:**

Neighborhood social and built environment characteristics may promote physical activity, a modifiable factor for dementia risk, which may in turn promote cognitive and brain health. We explored the associations of the U.S. National Walkability Index ‐ a nationwide geographic data resource that ranks block groups according to their relative walkability – with cognitive function, gray matter (GM) and white matter hyperintensities (WMH), neighborhood disadvantage, sedentary behavior and frequency of physical activity in cognitively unimpaired older adults at increased Alzheimer's disease (AD) risk.

**Method:**

We included baseline data from 1398 older adults (68.3 ± 5.17 years, 68.3% females, 30.5% APOE4 carriers) from the U.S. POINTER trial. Walkability was classified as: least, below average, above average and most walkable. Neighborhood disadvantage (low, moderate and high) was assessed using Area Deprivation Index (ADI) (*N* = 1384). Physical inactivity was assessed using the Sedentary Behavior Questionnaire (SBQ, hours sitting/week, *N* = 1106), and physical activity was measured with a composite score from the Community Health Activities Model Program for Seniors (CHAMPS) questionnaire. Linear and logistic regressions examined associations between walkability and (1) physical activity and sedentary behavior; and (2) cognition assessed with the Preclinical Alzheimer's Cognitive Composite (PACC‐5), GM volume and WMHs, Additionally, associations between neighborhood disadvantage and physical activity and sedentary behavior were explored. Models were adjusted for age, sex, race, APOE4 and total intracranial volume.

**Result:**

More deprived areas were generally less walkable (β = ‐0.008, *p* < 0.001) (Figure 1). Higher walkability (Above average: β = 0.192, *p* = 0.011 and most walkable: β = 0.183, *p* = 0.032) was associated with higher PACC‐5 (Figure 2A) scores but not with more preserved GM or lower WMH. Walkability scores were not associated with physical activity and sedentary measurements, while greater neighborhood disadvantage was linked to sedentary behavior (β = 0.126, *p* < 0.001) (Figure 2B).

**Conclusion:**

These results suggest that physical inactivity in more deprived areas is associated with neighborhood socioeconomic disadvantages rather than built environment. Additionally, walkability scores were not associated with any measure of physical activity or brain health, suggesting the need to further investigate other health effects of neighborhood disadvantage and walkability.